# Constitutive Activation of *AKT2* in Humans Leads to Hypoglycemia Without Fatty Liver or Metabolic Dyslipidemia

**DOI:** 10.1210/jc.2017-00768

**Published:** 2017-05-23

**Authors:** Marina Minic, Nuno Rocha, Julie Harris, Matthijs P. Groeneveld, Sarah Leiter, Nicholas Wareham, Alison Sleigh, Pascale De Lonlay, Khalid Hussain, Stephen O’Rahilly, Robert K. Semple

**Affiliations:** 1 The University of Cambridge Metabolic Research Laboratories, Wellcome Trust-Medical Research Council (MRC) Institute of Metabolic Science, Cambridge CB2 0QQ, United Kingdom; 2 The National Institute for Health Research, Cambridge Biomedical Research Centre, Cambridge CB2 0QQ, United Kingdom; 3 MRC Epidemiology Unit, University of Cambridge School of Clinical Medicine, Cambridge CB2 0QQ, United Kingdom; 4 Wolfson Brain Imaging Centre, University of Cambridge School of Clinical Medicine, Cambridge Biomedical Campus, Cambridge CB2 0QQ, United Kingdom; 5 National Institute for Health Research/Wellcome Trust Clinical Research Facility, Cambridge University Hospitals National Health Service Foundation Trust, Cambridge Biomedical Campus, Cambridge CB2 0QQ, United Kingdom; 6 Université Paris Descartes, Sorbonne Paris Cité, Faculté de Médecine, 75270 Paris Cedex 06, France; 7 Centre de Référence des Maladies Héréditaires du Métabolisme, Hôpital Necker, Assistance Publique-Hôpitaux de Paris, 75015 Paris, France; 8 Institut Imagine, Institut National de la Sante et de la Recherche Médicale, Unité 1163, 75015 Paris, France; 9 Department of Pediatric Medicine, Sidra Medical and Research Center, PO Box 26999, Doha, Qatar

## Abstract

**Context:**

The activating p.Glu17Lys mutation in AKT2, a kinase mediating many of insulin’s metabolic actions, causes hypoinsulinemic hypoglycemia and left-sided hemihypertrophy. The wider metabolic profile and longer-term natural history of the condition has not yet been reported.

**Objective:**

To characterize the metabolic and cellular consequences of the AKT2 p.Glu17Lys mutation in two previously reported males at the age of 17 years.

**Design and Intervention:**

Body composition analysis using dual-energy X-ray absorptiometry, overnight profiling of plasma glucose, insulin, and fatty acids, oral glucose tolerance testing, and magnetic resonance spectroscopy to determine hepatic triglyceride content was undertaken. Hepatic *de novo* lipogenesis was quantified using deuterium incorporation into palmitate. Signaling in dermal fibroblasts was studied *ex vivo*.

**Results:**

Both patients had 37% adiposity. One developed hypoglycemia after 2 hours of overnight fasting with concomitant suppression of plasma fatty acids and ketones, whereas the other maintained euglycemia with an increase in free fatty acids. Blood glucose excursions after oral glucose were normal in both patients, albeit with low plasma insulin concentrations. In both patients, plasma triglyceride concentration, hepatic triglyceride content, and fasting hepatic *de novo* lipogenesis were normal. Dermal fibroblasts of one proband showed low-level constitutive phosphorylation of AKT and some downstream substrates, but no increased cell proliferation rate.

**Conclusions:**

The p.Glu17Lys mutation of AKT2 confers low-level constitutive activity upon the kinase and produces hypoglycemia with suppressed fatty acid release from adipose tissue, but not fatty liver, hypertriglyceridemia, or elevated hepatic *de novo* lipogenesis. Hypoglycemia may spontaneously remit.

## Teaser

Two obese 17-year-old patients with genetic activation of AKT2 showed no fatty liver nor metabolic dyslipidemia, with persisting hypoglycemia and free fatty acid suppression in one.

In 2004, a single infant was described with a unique syndrome encompassing severe, persistent hypoketotic hypoglycemia despite replete, mobilizable hepatic glycogen stores and marked left-sided hemihyperplasia (1). Although the biochemical profile mimicked that of hyperinsulinism, no detectable insulin nor insulin-like substances were detectable at the time of hypoglycemia, and glucose infusion rates required to maintain euglycemia were much lower than are required in *bona fide* hyperinsulism. In 2011, we identified the *de novo* p.Glu17Lys mutation in AKT2 as the cause of this syndrome in the initial proband as well as in two further patients with the same metabolic disorder (2).

AKT2 is a serine/threonine kinase that plays a critical role in transducing insulin stimulation into metabolic responses in target tissues, including liver, adipose tissue, and skeletal muscle. Its activation is normally tightly coupled to stimulation of the insulin receptor by a requirement for phosphatidylinositol-3,4,5-trisphosphate (PIP3) for its recruitment to the plasma membrane. The p.Glu17Lys mutation relaxes this requirement, permitting binding to the more abundant membrane phospholipid phosphatidylinositol-4,5-bisphosphate (PIP2), partially uncoupling AKT2 activation from insulin stimulation (3, 4).

Since elucidation of the genetic cause of the syndrome, two further individuals with the same mutation, and with a similar biochemical profile, have been described (5, 6), with one report placing emphasis on dysmorphic features of the syndrome not previously noted (6). Moreover, studies using genome editing in stem cell–derived adipocytes and hepatocytes have corroborated the basal activation of the p.Glu17Lys mutation in AKT2 and have demonstrated activation of critical downstream responses in target cells, including glucose uptake in adipocytes and nuclear exclusion of the gluconeogenic transcription factor FOXO1 in hepatocyte-like cells (7).

Nevertheless, despite this body of evidence linking AKT2 p.Glu17Lys to hypoketotic hypoglycemia, key questions remain about the pathogenesis and natural history of the syndrome. Most critically from a clinical point of view, it is not yet known whether the tendency to hypoglycemia varies during puberty or when linear growth is completed, whereas from a physiological point of view, the relative contribution of AKT2 activation in liver and adipose tissue to hypoglycemia remains to be fully established. It is also not known whether genetic AKT2 activation in humans leads to accumulation of liver fat or metabolic dyslipidemia, which is an important question, as murine studies have strongly implicated AKT2 in the pathogenesis of fatty liver and related diseases associated with insulin resistance and hyperinsulinemia (8).

The aim of this study was to assess in more detail the metabolic phenotype in two of the patients previously reported, both male and, at the time of study, both 17 years old. Moreover, using dermal fibroblasts isolated from one patient, we aimed to characterize further the cellular phenotype associated with AKT2 p.Glu17Lys.

## Patients and Methods

All physiological studies were approved by the National Health Service Research Ethics Committee of the United Kingdom. All participants provided written informed consent, and all studies were conducted in accordance with the principles of the Declaration of Helsinki. Patients reserved the right to withdraw consent at any time.

### In vivo studies

Body composition was assessed using Lunar Prodigy dual-energy X-ray absorptiometry (GE Lunar). Hepatic triglyceride was assessed using proton magnetic resonance spectroscopy on a Siemens 3T Verio scanner using methods previously described (9) and quantified as the ratio of methylene to combined methylene and water signals corrected for spin-spin relaxation. Adipose distribution was determined by magnetic resonance imaging using T_1_-weighted turbo spin echo, water-suppressed, transaxial images, also generated by a Siemens 3T Verio scanner and pseudocolored for visualization using MATLAB software (The MathWorks, Inc., Natick, MA). The fractional *de novo* synthetic rate of fatty acids was determined by assessing the incorporation of deuterium into plasma triglyceride after the administration of deuterium-labeled water. After a balanced meal of standard macronutrient composition at 7 pm, deuterium-labeled water (3 mL/kg body water, 99.8%; Cambridge Isotopes) was administered in two doses of equal size (8 pm, 10 pm). Blood was collected before and 12 hours after the first loading dose of deuterium. Deuterium enrichment of plasma palmitate was determined by gas chromatography/mass spectrometry (5973N system; Agilent, Santa Clara, CA) and compared with that of water, determined by isotope ratio mass spectrometry using a SIRA10 instrument (VG Isotopes Ltd). A reference range was determined from nine healthy volunteers (9, 10).

For overnight metabolic profiling, participants were admitted to a clinical research facility and fasted from 10 pm. Samples were taken hourly for biochemical analysis overnight, or in the event of symptomatic hypoglycemia. In one participant, hypoglycemic symptoms developed after 2 hours of fasting, with a blood glucose of 48 mg/dL, and 200 mL of oral Lucozade^TM^ was given containing 34 g glucose. Seventy-five-gram oral glucose tolerance testing was undertaken in both study subjects at 8 am following the overnight fast, with blood sampling at the times indicated.

### Biochemical assays

For measurement of insulin, leptin, and adiponectin, venous blood was drawn in the fasting state and plasma was immediately extracted and stored at –20°C. Proteins were assayed using two-step, time-resolved AutoDELFIA immunoassays, in accordance to the previously described protocols (9, 11, 12). Normative adiponectin, leptin, and oral glucose tolerance testing data were derived from a white population from the Medical Research Council Ely Study cohort, representative of an ethnically homogeneous white population in this area of eastern England (11, 13). Reference data for lipid parameters were derived from 42 healthy volunteers [women: n = 23, mean age, 36 years (range, 23 to 62), mean body mass index (BMI), 23.5 kg/m^2^ (range, 19.1 to 32.7); men: n = 19, mean age, 44 years (range, 22 to 62), mean BMI, 25.5 kg/m^2^ (range, 18.4 to 31.7)] (9). All other biochemical analyses were undertaken in an accredited clinical diagnostic laboratory in Addenbrooke’s Hospital, Cambridge, United Kingdom.

### Cellular signaling studies

Human primary dermal fibroblasts from healthy controls and patient 1 were obtained and cultured using standard procedures (1). Cell lines were confirmed prior to study by genotyping of AKT2. Cells were grown to 90% confluence prior to serum starvation. Serum-free culture medium consisted of high-glucose Dulbecco’s modified Eagle medium supplemented with 100 U/L penicillin-streptomycin, 100 μg/mL l-glutamine, and 0.5% bovine serum albumin. Cells were washed with warm phosphate-buffered saline and serum starved for 18 to 24 hours before stimulation. After 18 to 24 hours, serum-free medium was removed and replaced with serum-free medium containing the appropriate concentration of insulin (Actrapid; NovoNordisk, Crawley, United Kingdom), IGF-1 (Abcam, Cambridge, United Kingdom), or EGF (Sigma-Aldrich, Gillingham, United Kingdom) for 5 or 10 minutes. Cells were then washed with cold phosphate-buffered saline, snap frozen in liquid nitrogen, and thawed and scraped in modified radioimmunoprecipitation assay buffer (substitution of 1% [volume-to-volume ratio] Triton-X 100 instead of sodium dodecyl sulfate) containing Complete Mini Protease Inhibitor Cocktail (Roche, Burgess Hill, United Kingdom). Protein concentrations were determined with the Bradford protein assay (Bio-Rad).

Phosphorylation of AKT in cell lysates was assessed using InstantOne ELISA Kits (eBioscience, Inc., San Diego, CA), according to the manufacturer’s guidelines. The PathScan® Akt Signaling Antibody Array Kit (Chemiluminescent Readout; Cell Signaling Technology, Danvers, MA) was used for simultaneous assessment of 16 phosphorylated proteins within the AKT signaling pathway. All reagents and the experimental protocol were as provided by the manufacturer. An image of the slide was captured with a Bio-Rad ChemiDoc MP Digital Imager and analyzed using AlphaView array analysis software (ProteinSimple, Santa Clara, CA).

For immunoblotting studies, sodium dodecyl sulfate–polyacrylamide gel electrophoresis was undertaken followed by transfer to a polyvinylidene difluoride membrane using the iBlot dry blotting system (Thermo Fisher Scientific, Cramlington, United Kingdom) and immunoblotting with the appropriate primary antibodies. Secondary antibodies were either horseradish peroxidase linked, in which case, proteins were visualized using enhanced chemoluminescence (GE Health Care, Little Chalfont, United Kingdom; Millipore, Billerica, MA), or IRDye® fluorophore linked, in which case an Odyssey IR Imaging System (LI-COR Bioscience, Lincoln, NE) was used. Western blot band intensity was quantified by densitometry and analyzed using ImageJ software, with intensity of bands of interest normalized to the intensity of the corresponding loading control (calnexin, unless indicated otherwise). Primary antibodies used are indicated in the Supplemental Methods.

### Statistical analysis

Statistical significance of differences in protein phosphorylation was analyzed using the two-tailed, unpaired Student *t* test. Differences in cell proliferation rates were assessed by fitting linear regression lines and then testing for gradient equality. Significance is shown on all graphs: * denotes *P* < 0.05, ** denotes *P* < 0.01, *** denotes *P* < 0.001, and nonsignificant indicates *P* > 0.05. All bars represent mean ± standard error of the mean, unless otherwise indicated. All analyses were performed in GraphPad Prism 5.0 (GraphPad Software Inc., San Diego, CA).

## Results

### Physiological study of two affected probands

At the age of 17 years old, two male patients [designated patient 1 (P1) and patient 2 (P2)] whose early clinical courses were previously described (2) were reassessed. Both participants and their families remained very anxious about hypoglycemia; however, the severity of this had lessened since early life. P1 still had a percutaneous feeding tube *in situ* and was receiving overnight feeding; however, he was well and active and able to engage in sport. Although the feeding tube had been removed in P2 at the age of 11 years, he was only able to maintain euglycemia using three hourly feeds as well as an additional bedtime feed with modified maize starch. He reported significant weakness and easy fatiguability on exertion.

**Figure 1.**
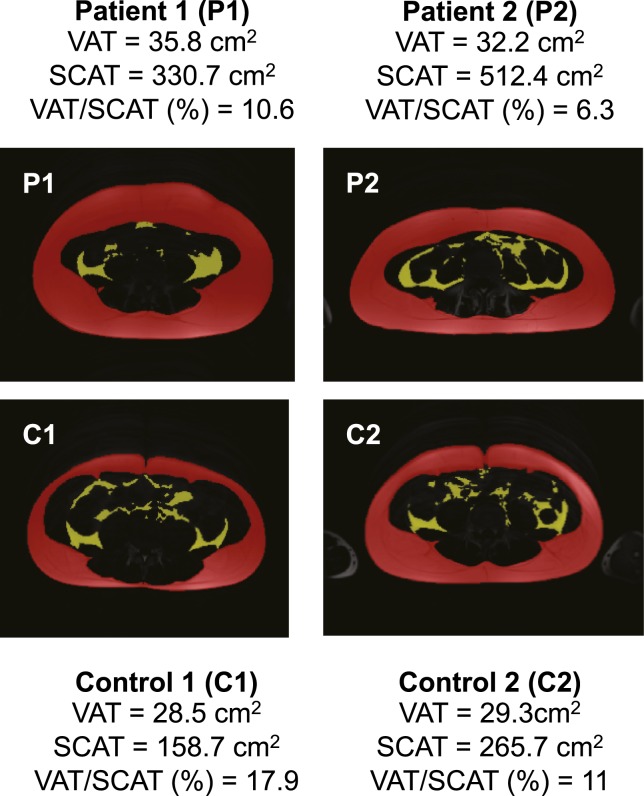
Quantification of visceral and subcutaneous adipose tissue. Representative abdominal fat distribution determined by magnetic resonance imaging at the L4 vertebral level is shown with pseudocolored adipose depots for P1, P2, and two healthy sex- and age-matched controls (C1, C2). Visceral adipose tissue (VAT) is indicated in yellow, and subcutaneous adipose tissue (SCAT) is indicated in red.

On examination, both P1 and P2 had generalized obesity. Cross-sectional imaging demonstrated particularly marked expansion of subcutaneous adipose tissue, with visceral fat accounting for only 10.6% and 6.3% of fat in P1 and P2, respectively (Fig. 1). Corresponding values in 12 healthy male volunteers [mean age, 15.7 years (range, 14.6 to 16.9); mean BMI, 20.2 (range, 16.5 to 23.5); total % adiposity, 15.0 (range, 7.0 to 30.8)] were 11.0% to 45.8% (mean, 24.2%). Both patients were also noted to have mild bilateral ptosis with proptosis, in keeping with the dysmorphic features described in two previous patients with the AKT2 p.Glu17Lys mutation (5, 6). Previously noted left-sided hemihyperplasia remained striking in P1, affecting subcutaneous adipose tissue in particular, but was only subtle and limited to the face in P2. Body composition analysis showed both P1 and P2, coincidentally, to have an identical body adipose content of 37%.

**Figure 2.**
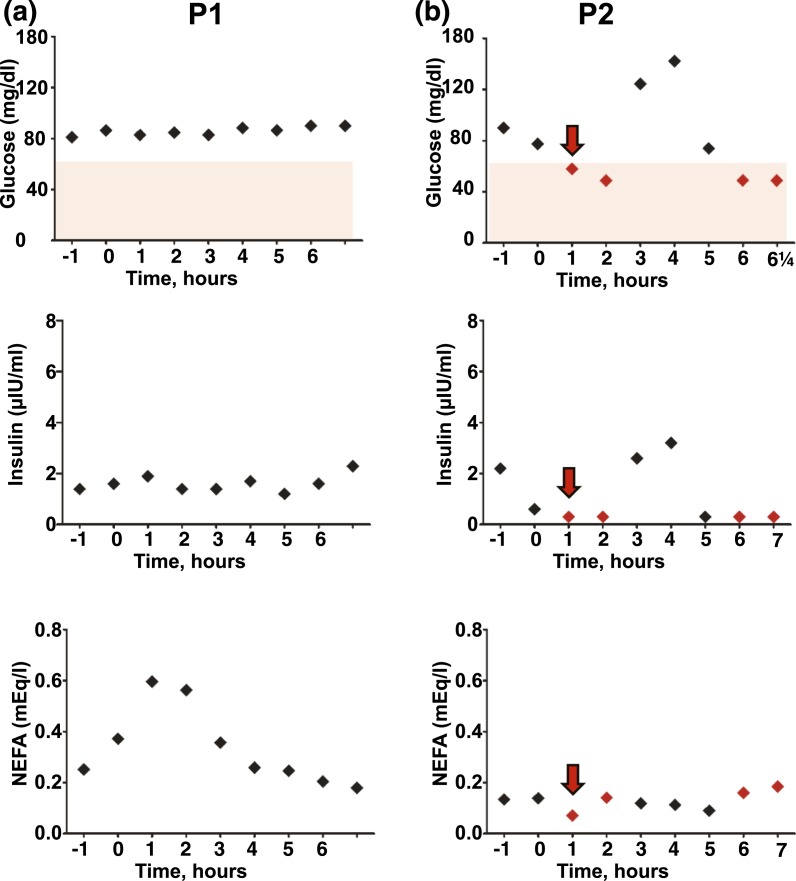
Overnight profiling of plasma glucose, insulin, and free fatty acid concentrations. Plasma concentrations of glucose, insulin, and free fatty acids are shown during fasting from 11 pm for (a) P1 and for (b) P2. Midnight is represented by 0 hours. Samples taken during spontaneous hypoglycemia in P2 are indicated with red icons, whereas administration of an oral “rescue” bolus of 34 g glucose solution is indicated by red arrows. NEFA, nonesterified fatty acids.

To evaluate fasting tolerance, P1 and P2 underwent an overnight fast with hourly blood sampling. P1 remained euglycemic, with a physiologically appropriate increase in free fatty acids overnight, but with notably low plasma insulin concentration throughout the night [Fig. 2(a)]. P2, in contrast, developed symptomatic hypoglycemia after 3 hours of fasting, with suppression of both plasma insulin and free fatty acids, requiring rescue with a bolus of oral glucose solution containing 34 g glucose, following which no further intervention was required [Fig. 2(b)]. Plasma free fatty acids remained low throughout the night.

**Figure 3.**
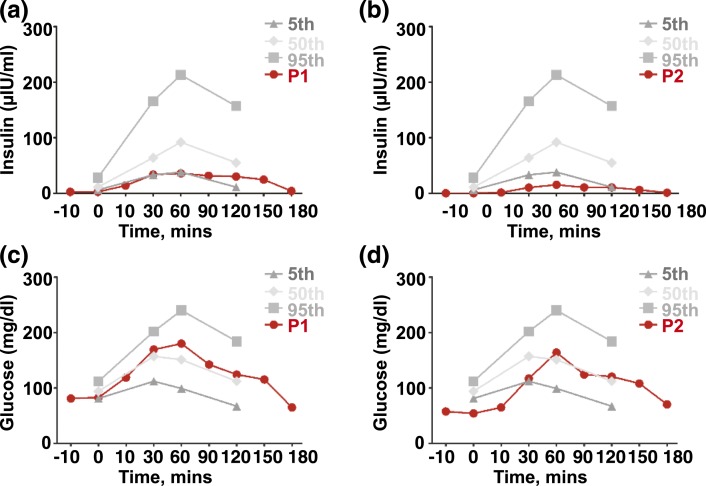
Oral glucose tolerance testing. Plasma glucose and insulin concentrations after a 75-g oral glucose load are shown for (a and b) P1 and (c and d) P2. Testing was undertaken following the overnight fast illustrated in Fig. 2. Sex-matched control data from a large European white control population from eastern England are shown as 5th, 50th, and 95th percentiles.

Overnight fasts were immediately followed by oral glucose tolerance testing to assess the response to a glucose challenge. In P1, fasting glucose values were within the normal reference range, whereas P2 was hypoglycemic at termination of the overnight fast. After the glucose challenge, however, glucose levels in both P1 and P2 exceeded the 50th percentile within 60 minutes, although concomitant insulin excursion tracked below the 50th percentile and 5th percentile in P1 and P2, respectively, with reference to a large population-based control data set (Fig. 3).

Fasting plasma triglyceride concentrations were normal for both P1 and P2; however, in both participants, fasting plasma very low-density lipoprotein (VLDL) cholesterol and high-density lipoprotein (HDL) cholesterol concentrations were low. Proton magnetic resonance spectroscopy revealed normal hepatic triglyceride content, with an intrahepatic lipid-to-water ratio of 0.7% and 0.1% in P1 and P2, respectively (reference range from healthy controls <5.5%). Moreover, there was no difference in the rate of fasting *de novo* lipogenesis when compared with healthy controls (Table 1). Serum leptin levels were increased in both participants, consistent with their elevated adiposity, whereas serum adiponectin concentrations were at or above the top of a reference range derived from BMI- and sex-matched controls.

**Table 1.** **Biochemical Profile of Patients 1 and 2 With the AKT2 p.Glu17Lys Mutation at 17 Years Old**

	**P1**	**P2**	**Reference Range**
Age	17	17	—
BMI (kg/m^2^)	28.6	22	<25
Glucose (mg/dL)	81	54	<110
Insulin (µIU/mL)	2.7	<0.3	0–8.6
C-peptide (ng/mL)	0.7	0.05	0.5–2.7
Leptin (ng/mL)	45.8	113	1.5–13*^a^*
Adiponectin (mg/L)	10.1	14.9	P1: 2.4–10.6*^a^*
			P2: 2.6–12.6*^a^*
NEFA (mEq/L)	0.43	0.3	0.2–0.9
Cholesterol, total (mg/dL)	131	112	205–228
HDL cholesterol (mg/dL)	31	41	53–66
LDL cholesterol (mg/dL)	89	66	<116
VLDL Tg (mmol/L)	27.4	18.6	40–61
Triglyceride (mg/dL)	60	42	102–144
Hepatic triglyceride (%)	0.7	0.1	<5.5%
Hepatic DNL (%)	1.9	1.8	<2.5%

Blood was drawn after an overnight fast interrupted by a single bolus of oral glucose in P2, as described in text.

Abbreviations: DNL, *de novo* lipogenesis; LDL, low-density lipoprotein; NEFA, nonesterified fatty acids; Tg, triglyceride.

^a^ BMI- and sex-adjusted reference values.

Limited evaluation of both parents of P1 was also undertaken. Both were obese, and the father of P1 had marked flexural acanthosis nigricans. Biochemical analysis also revealed the father to have fasting hyperinsulinemia with normal fasting glucose and suppressed HDL cholesterol, although other analytes fell within the reference range. The mother of P1 was also obese, and insulin and C-peptide were at the upper limit of the reference range, with normal fasting glucose. The lipid profile was normal (Supplemental Table 1).

### Primary fibroblast signaling studies

**Figure 4.**
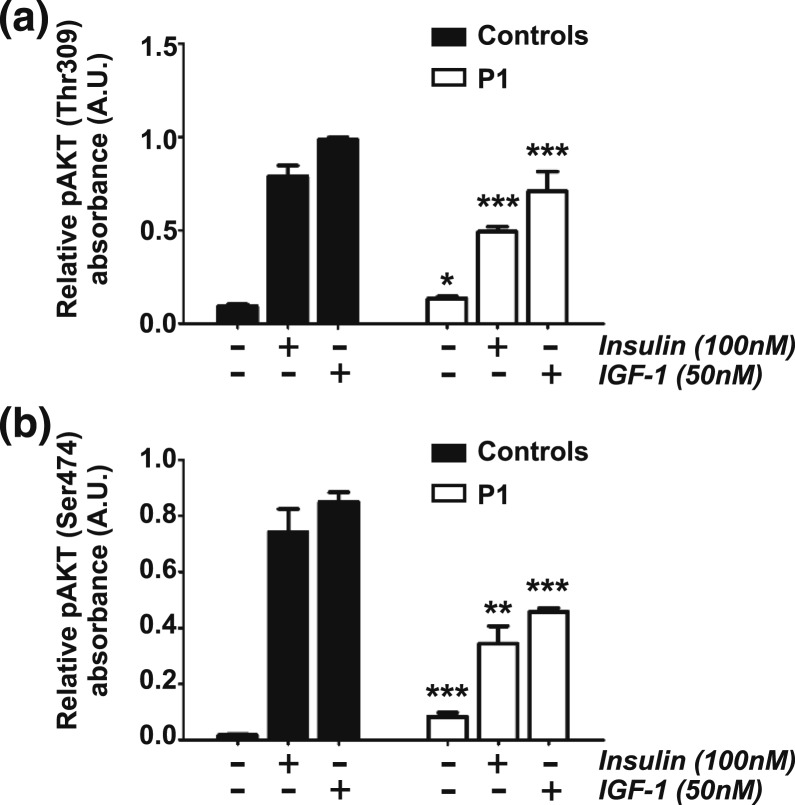
Insulin and IGF-1 signaling in primary dermal fibroblasts from P1. Phosphorylation of AKT at (a) threonine 308/309 (Thr309 in AKT2) and (b) serine 473/474 (Ser474 in AKT2) in dermal fibroblasts from P1 and three healthy controls assessed after 18 hours of serum starvation with or without insulin or IGF-1 stimulation. Results shown are means of three independent experiments, with error bars representing standard deviations. Differences in protein phosphorylation between patient cells and the corresponding control cells under the same conditions of ligand stimulation were tested by unpaired *t* test. *P < 0.05, **P < 0.01, ***P < 0.001. A.U., arbitrary units.

Previous studies established a low level of basal hyperphosphorylation of AKT in primary fibroblasts from P1. This finding was extended in the same dermal fibroblasts compared with dermal fibroblasts obtained from four healthy volunteers. Low-level basal phosphorylation of AKT was confirmed, though, surprisingly, peak phosphorylation of AKT after stimulation of cells with insulin or IGF-1 was blunted in P1 cells compared with controls (Fig. 4). Using a commercially available immunochip to assay other components of the PI3K/signaling pathway confirmed patchy activation of pathways downstream from AKT, with PRAS40 phosphorylation particularly robust (Supplemental Fig. 1). No difference in cell proliferation rate between P1 and control dermal fibroblasts could be discerned, however, either in serum-replete or serum-starved conditions (Supplemental Fig. 2).

Additional analyses focused on expression of components of the INSR/IGF-1R and EGF signaling pathway, as several of those are subject to negative feedback after activation of PI3K/AKT signaling. In P1 cells relative to healthy controls, there was a decrease in IGF1R, EGFR, and IRS2 protein levels; however, there were no changes in IRS1, INSR, p85*α*, or p110*α* protein levels, the last two proteins together comprising a key isoform of type 1A PI3K that lies upstream in the signaling pathway from AKT (Supplemental Fig. 2). These findings illustrate the remodeling of RTK/PI3K/AKT signaling that occurs in response to tonic low-level activation of AKT2 even in noncanonically insulin-responsive cells.

## Discussion

Aninsulinemic hypoglycemia due to genetic activation of AKT2 is a rare entity, described to date in only five patients. Nevertheless, the severity and persistence of the hypoglycemia dominates the lives of affected children and their families, who have a pressing need to understand the natural history of the condition. Moreover, the critical importance of AKT2 not only in mediating key actions of insulin in health, but also potentially in driving components of the metabolic syndrome such as fatty liver and dyslipidemia in the face of insulin resistance, lends considerable interest to the wider metabolic observations to be made in this rare human “experiment of nature.”

Our first significant clinical observation is that one patient at the age of 17 years old is able to sustain a normal blood glucose overnight, with a normal increase in plasma free fatty acids during the night. The second patient, of the same sex, age, and body composition, in contrast, has a fasting tolerance of only 2 to 3 hours and shows suppressed free fatty acid levels throughout the night. At present, it is not clear whether the propensity to hypoglycemia of P2 will also lessen in due course, nor what the mechanism underlying diminution of the metabolic disturbance in P1 is. It is notable that the father of P1 shows evidence of obesity-related insulin resistance. No similar parental studies were possible for P2; however, this finding raises the possibility that, in P1, progressive obesity has led to a state of insulin resistance determined by inherited, presumably oligogenic factors and that this is exerted at or beyond the level of AKT2 in liver and adipose tissue, effectively attenuating the basal AKT activation that was driving hypoglycemia and free fatty acid suppression.

One proband reporting previously with the loss-of-function p.Arg274His mutation in AKT2 showed femorogluteal lipodystrophy (14), whereas both patients whom we describe at 17 years old were obese. Moreover, in murine studies, Akt2 has been suggested to play a critical role in the expansion of visceral adipose tissue on exposure of mice to a high-fat diet, and the previous report of obesity in patients with the AKT2 p.Glu17Lys mutation has been suggested to support an important cell autonomous role of AKT2 in adipocyte development (15). However, evidence that Akt2 activation is necessary for adipose tissue expansion does not also establish that it is sufficient, and a major confounder in the present patients is chronic caloric excess, engendered by the need to avoid prolonged fasting and exacerbated by a desire by both physicians and patients or their families to err on the side of caution with supplemental feeding. It is notable that adipose expansion in the patients we describe was largely in subcutaneous depots, rather than being predominantly visceral, as might be expected if AKT2 played a preferential role in mediating expansion of visceral adipose tissue.

Several lines of evidence suggest that hyperinsulinemia contributes significantly to the fatty liver and dyslipidemia seen in common insulin-resistant states. Both patients studied here had normal liver triglyceride content, normal or low plasma triglyceride concentrations, and normal rates of fasting *de novo* lipogenesis despite low-level constitutive activation of AKT2 and obesity. Conversely, previously described patients with a loss-of-function mutation in AKT2 had insulin resistance, with severe fatty liver and dyslipidemia, quite unlike patients with severe insulin resistance due either to mutations in the insulin receptor (9), who exhibit normal hepatic lipid content, normal rates of fasting *de novo* lipogenesis, and a normal plasma lipid profile despite severe insulin resistance, or to mutations in PIK3R1, encoding a regulatory subunit of PI3K, who also show a normal lipid profile despite severe insulin resistance (16).

One interpretation of these observations is that AKT2 is *not* required for hepatic *de novo* lipogenesis in humans. Instead, *de novo* lipogenesis might be dependent on a branch of the insulin signaling pathway that is downstream of the insulin receptor and PI3K, but upstream from AKT2. In support of this view are two murine studies that suggest that PKC, and not AKT, contributes to the increase in Srebp-1c expression (17, 18). Contrary to these reports, several other groups suggest that mTORC1 and AKT2 are positive regulators of *de novo* lipogenesis (8, 19, 20). Caution should be exercised, however, in interpreting these human findings solely in terms of the role of AKT2 in the liver. AKT2 is also expressed in adipose tissue and skeletal muscle, and it is highly plausible that effects of genetically activated AKT2 in adipose tissue are masking any propensity toward accumulation of liver fat. Indeed, the clear suppression of normal overnight increases in fatty acids in one of the patients studied is most easily explicable by pathological suppression of lipolysis in adipocytes due to tonic AKT2 action. This would have the effect of reducing one major source of liver triglyceride, while simultaneously depriving the liver of substrate for *β* oxidation and energy generation, which has been shown to be important for gluconeogenesis in murine studies (21). Reduction in free fatty acid delivery to the liver may explain the low VLDL cholesterol levels recorded in the patients described.

The cellular signaling studies we describe illustrate that the p.Glu17Lys mutation does not confer full activation of AKT2, but rather a low level of inappropriate basal activation. Indeed, in the single patient cell line studied, peak phosphorylation in response to a panel of growth factors was blunted, likely due to extensive feedback degradation or suppression of expression of proximal signaling molecules, including receptors and insulin receptor substrates. It is not clear, however, whether this cellular phenomenon has any *in vivo* correlate. The level of AKT activation seen appears sufficient to activate some but not all downstream phosphorylation events. This observation is in agreement with cellular evidence that AKT serves to amplify and transmit input signals into outputs in a nonlinear manner (22) with different clusters of AKT substrates identifiable based on their capacity to be phosphorylated by the different concentrations of insulin (23). This may also explain why much less glucose is required to maintain euglycemia in infants with AKT2 p.Glu17Lys, in which the metabolic defect may be failure to derepress gluconeogenesis during fasting, than in those with hyperinsulinism, in whom there is also inappropriate disposal of glucose into skeletal muscle and adipose tissue mediated by GLUT4.

Our findings suggest that hypoglycemia driven by the AKT2 p.Glu17Lys mutant may not be lifelong and may remit under some circumstances, although whether developmental, genetic, or environmental modifying factors are most critical in this remains to be determined. More generally, we provide evidence for complexity in both the systemic and cellular consequences of low-grade, constant AKT2 activation. Detailed study of similar-graded perturbations of Akt2 in different mouse tissues is likely to be required to tease out the interrelationships between Akt2 activation and the phenotype we observe in humans.
