# Supplementary material for: Constitutive Activation of AKT2 in Humans Leads to Hypoglycemia Without Fatty Liver or Metabolic Dyslipidemia
Source: J Clin Endocrinol Metab. 2017 May 23;102(8):2914–21. doi: 10.1210/jc.2017-00768 (PMC5546860; doi:10.1210/jc.2017-00768)
Supplement: Supplementary file 1 [file jc.2017-00768.sd1.docx]

**
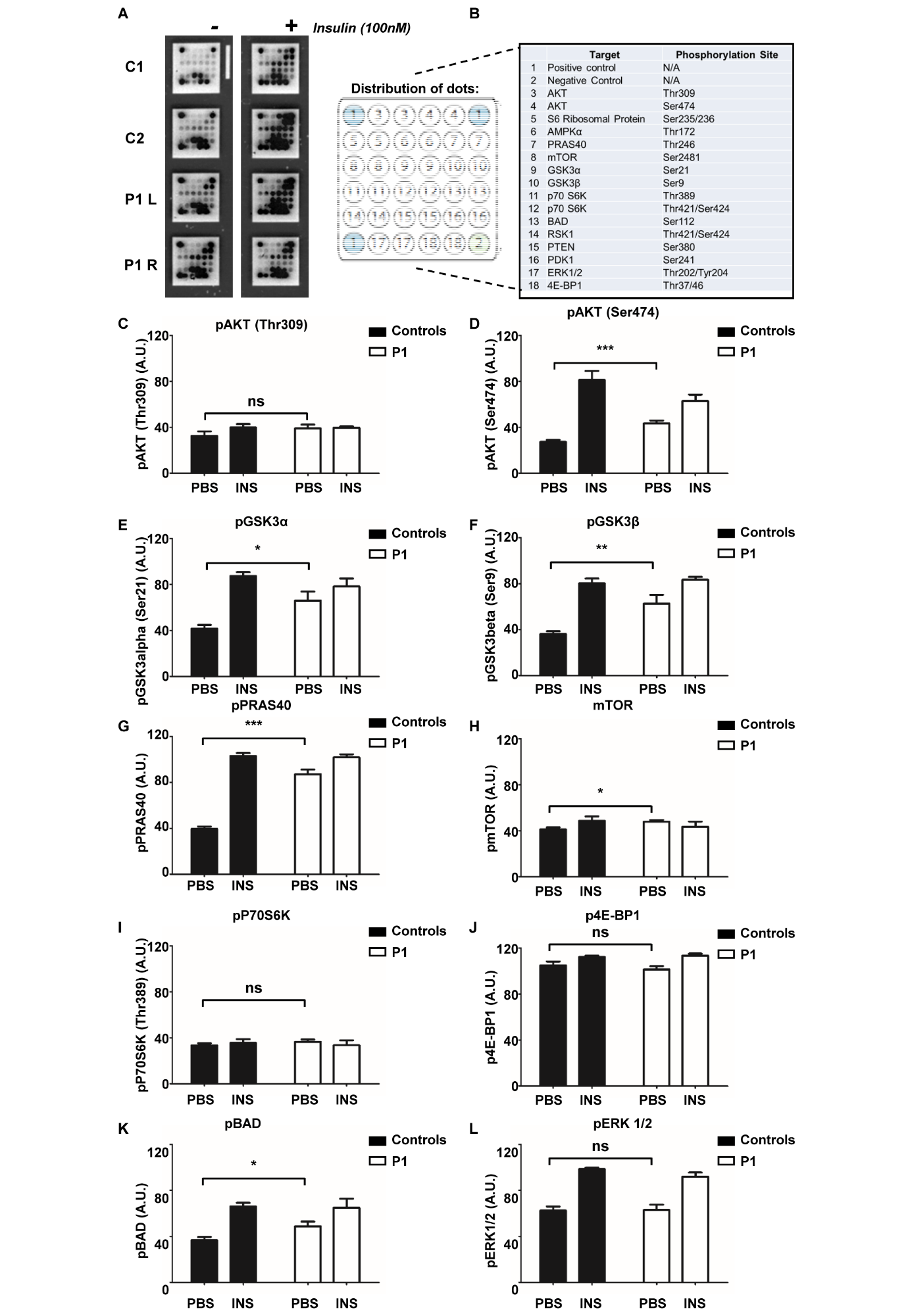
**

**Supplemental Figure S1: Analysis of the insulin signalling pathway using the PathScan® Akt Signalling Antibody Array Kit.** Cell lysates obtained from left and right side of Patient 1 (P1 L and P1 R) and two healthy controls were subjected to analysis using the PathScan® Akt Signalling Antibody Array Kit. Panel A shows image that was acquired using the BioRad imaging system, together with a table representing a distribution of dots corresponding to target-specific capture antibodies (panel B). Panels C-L show quantification of results obtained using the AlphaView Software. Results were normalised to an endogenous positive control. Healthy controls (black bars); P1 (white bars), unpaired t-test, *=p<0.05. A representative image of the two independent experiments is shown, error bars represent mean±SEM.


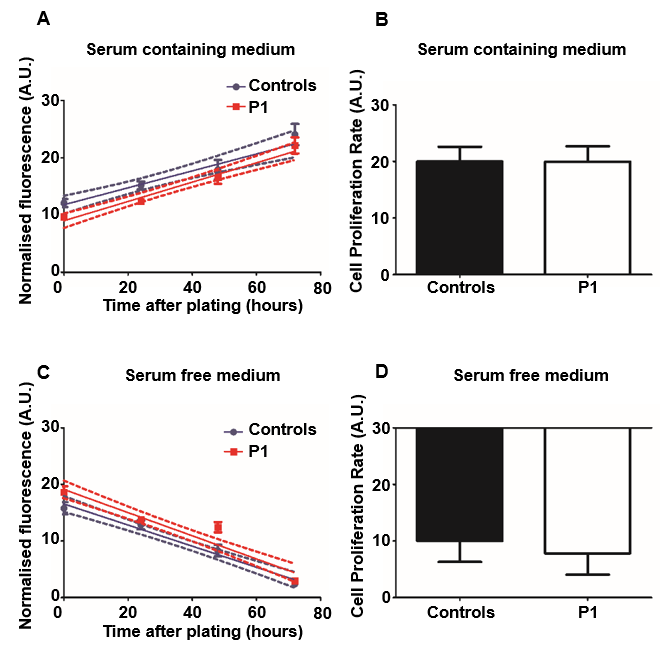


**Supplemental Figure S2. Growth rate of dermal fibroblasts from patient 1** The proliferation rate of dermal fibroblasts from four healthy control volunteers and Patient 1 (P1) was assessed in serum-containing (A,B) and serum-free medium (C,D). Representative data from one of three independent replicates are shown. Each data point in (A) and (C) represents mean ± standard deviation of 4-8 wells, and dotted lines represent 95% confidence intervals. Gradients were not significantly different between cell lines. No difference was found between rates of growth of control and P1 cells on testing with unpaired Student’s t-test, as shown in (B,D).

**
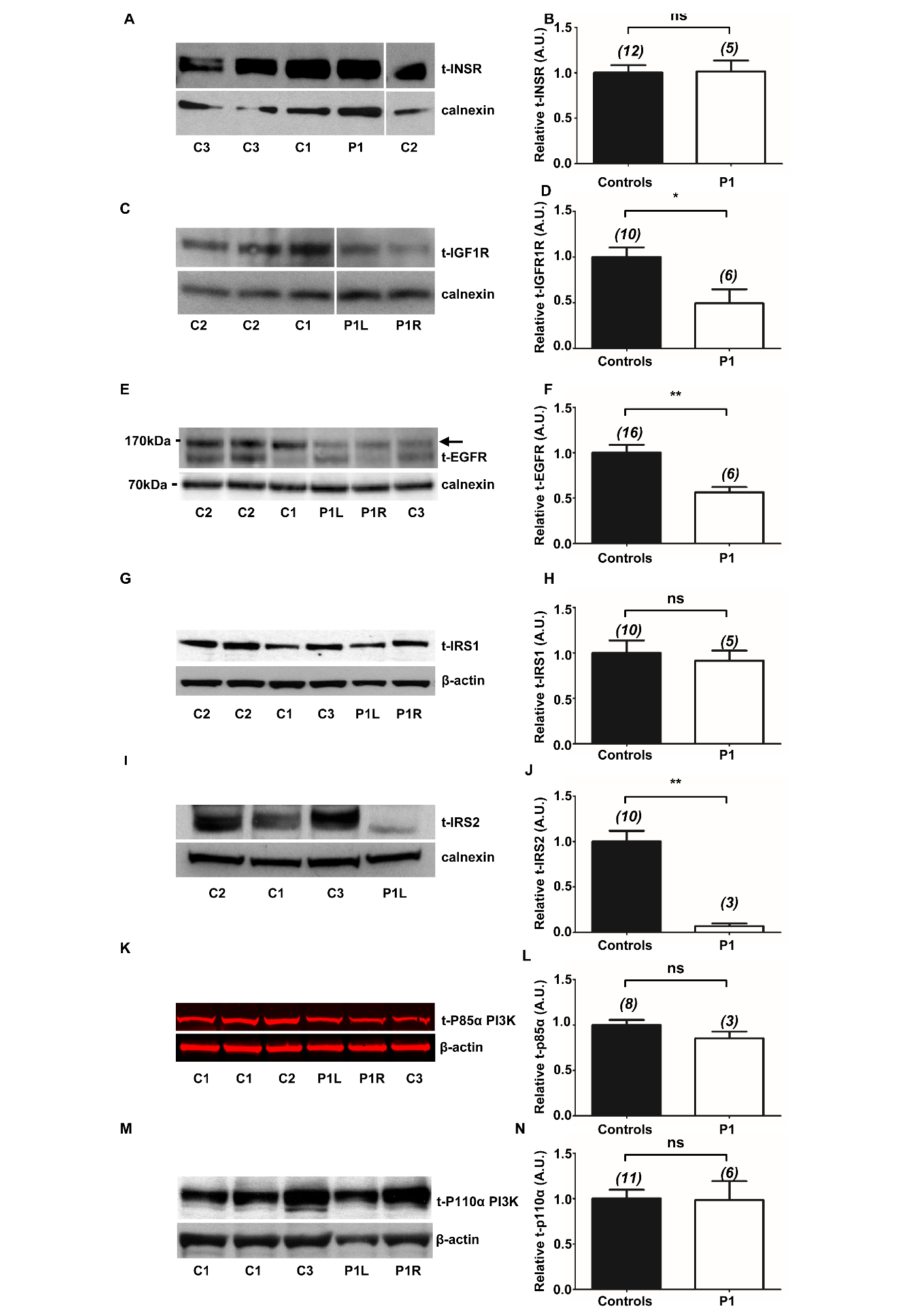
**

**Supplemental Figure S3: Total protein expression of components of the receptor tyrosine kinase/PI-3-Kinase/AKT signaling pathway in primary dermal fibroblasts**. Western blot analysis (left side) and corresponding quantifications (right side) are shown for serum-starved dermal fibroblasts for (A,B) Insulin receptor (INSR); (C,D) Insulin-like growth factor 1 receptor (IGF1R); (E,F) Epidermal Growth Factor Receptor (EGFR, (G,H); Insulin Receptor Substrate 1 (IRS1); (I,J) Insulin Receptor Substrate 2 (IRS2); (K,L) the p85α regulatory subunit of phosphatidylinositol 3-kinase; (M,N) the p110α catalytic subunit of phosphatidylinositol 3-kinase. All immunoblots have been obtained using antibodies specific to total protein levels (as indicated by the “t” in front of the protein names). P1 fibroblasts (left side P1L and right side P1R) are compared to three healthy negative control (C1-C3). Blots are representative of four (E, M), three (A, C, G, I) and two (K) independent experiments. Calnexin or β-actin was used as a loading control. Error bars in bar graphs represent mean±SEM. Gel images have been cut to remove irrelevant lanes where appropriate for presentation. In all cases composite images were taken from the same image of the same gel. The total number of individual measurements used to derive means and SEM are given in parenthesis. P1L and P1R values were analysed together. **<0.01, *p<0.05, ns – non significant by unpaired Student’s t-test.

**Supplemental tables**

|  | **P1’s**  **Father** | **P1’s**  **Mother** | **Fasting**  **reference range** |
| --- | --- | --- | --- |
| Age | 51 | 50 | -- |
| Height | 182 | 157 | -- |
| Weight | 113 | 89 | -- |
| BMI (kg/m2) | 34.1 | 36.1 | <25 |
| Glucose (mg/dl) | 83 | 101 | <110 |
| Insulin (µIU/ml) | 16 | 9 | 0-8.6 |
| C-peptide (ng/ml) | 5.1 | 2.8 | 0.5-2.7 |
| Leptin (ng/ml) | 18 | 65 | M: 4.2-26*  F: 22.7-113.6 |
| Adiponectin (mg/l) | 3.2 | 7.3 | M: 2.8-14.9*  F: 2.6-17.1 |
| NEFA (mEq/l) | 0.4 | 0.6 | 0.3-0.9 |
| Cholesterol, total (mg/dl) | 182 | 151 | 205-228 |
| HDL cholesterol (mg/dl) | 33 | 41 | 53-66 |
| LDL cholesterol (mg/dl) | 124 | 77 | <116 |
| Triglyceride (mg/dl) | 124 | 168 | 102-144 |

**Supplemental Table S1. Fasting biochemical profile of parents of patient 1.** *BMI- and sex-specific reference values

| Target protein | Species | Company | Catalogue No |
| --- | --- | --- | --- |
| *Primary antibodies* |  |  |  |
| INSR beta(C-19) | Rabbit | Santa Cruz Biotechnology, Santa Cruz, CA | sc-711 |
| IGF1-1R beta(C-20) | Rabbit | Santa Cruz Biotechnology, Santa Cruz, CA | sc-713 |
| EGFR (1005) | Rabbit | Santa Cruz Biotechnology, Santa Cruz, CA | sc-03 |
| IRS-1 | Rabbit | Cell Signalling Technology, Beverly, MA | 2382 |
| IRS-2 | Rabbit | Cell Signalling Technology, Beverly, MA | 4502 |
| PI3K p110alpha | Mouse | BD Biosciences, San Jose, CA | 611398 |
| PI3K p85 | Rabbit | Cell Signalling Technology, Beverly, MA | 4257 |
| Beta-actin | Rabbit | Abcam, Cambridge, UK | ab8229 |
| Calnexin | Rabbit | Abcam, Cambridge, UK | ab75801 |
| *HRP-linked Secondary antibodies* |  |  |  |
| Rabbit IgG (H+L) HRP-linked | Donkey | Thermo-Scientific/Pierce, Waltham, MA | 31458 |
| Mouse IgG HRP-linked | Goat | Cell Signalling Technology, Beverly, MA | 7076 |
| *LI-COR secondary antibodies* |  |  |  |
| IRDye® 680LT anti-Rabbit IgG | Goat | LI-COR Biotechnology -UK Ltd, Cambridge, UK | P/N 926-68021 |

**Supplemental Table S2. Antibodies for Western blotting**

| Target protein | Application | Manufacturer | Catalogue No |
| --- | --- | --- | --- |
| phospho-AKT 1/2/3 (Ser473) InstantOne™ ELISA | ELISA | eBioscience, Ltd. Hatfield, UK | 85-86042-11 |
| phospho-AKT 1/2/3 (Thr308) InstantOne™ ELISA | ELISA | eBioscience, Ltd. Hatfield, UK | 85-86044-11 |
| AKT 1/2/3 (Total) InstantOne ELISA | ELISA | eBioscience, Ltd. Hatfield, UK | 85-86045-11 |

**Supplemental Table S3. List of antibodies used for detection of protein phosphorylation by ELISA**
